# Cardiogenic shock due to yew poisoning rescued by VA-ECMO: case report and literature review

**DOI:** 10.1080/19336950.2022.2104886

**Published:** 2022-08-08

**Authors:** Nikolaus Schreiber, Martin Manninger, Sascha Pätzold, Alexander C. Reisinger, Stefan Hatzl, Gerald Hackl, Christoph Högenauer, Philipp Eller

**Affiliations:** a Department of Internal Medicine, Intensive Care Unit, Medical University of Graz, Graz, Austria; b Department of Internal Medicine, Division of Cardiology, Medical University of Graz, Graz, Austria; c Department of Internal Medicine, Division of Gastroenterology and Hepatology, Medical University of Graz, Graz, Austria

**Keywords:** ECMO, cardiogenic shock, taxine alkaloids, yew

## Abstract

Ingestion of leaves of the European yew tree (*Taxus baccata*) can result in fatal cardiac arrhythmias and acute cardiogenic shock. This cardiotoxicity derives from taxine alkaloids that block cardiac voltage-gated sodium and calcium channels. Prompt initiation of venoarterial extracorporeal membrane oxygenation is essential to bridge these critically ill patients to recovery, as there is no antidote available. We here report a 39-year old patient with toxic cardiogenic shock after yew poisoning, who was successfully rescued by venoarterial extracorporeal membrane oxygenation and had a full neurological recovery. This report emphasizes the role of intoxications as reversible causes of cardiac arrest and adds further evidence to the body of existing literature thus encouraging the early use of venoarterial extracorporeal membrane oxygenation in patients with yew poisoning and cardiogenic shock.

## Introduction

The potentially fatal effects of the European yew tree (*Taxus baccata*) were known since ancient times [1] and derive from taxine alkaloids that inhibit sodium and calcium channels in cardiomyocytes [2]. We report the case of a 39-year old male, who developed an acute cardiogenic shock after ingestion of multiple yew branches and leaves, and barely survived after prompt resuscitation with venoarterial extracorporeal membrane oxygenation (VA-ECMO).

## Case report

**Figure 1.**
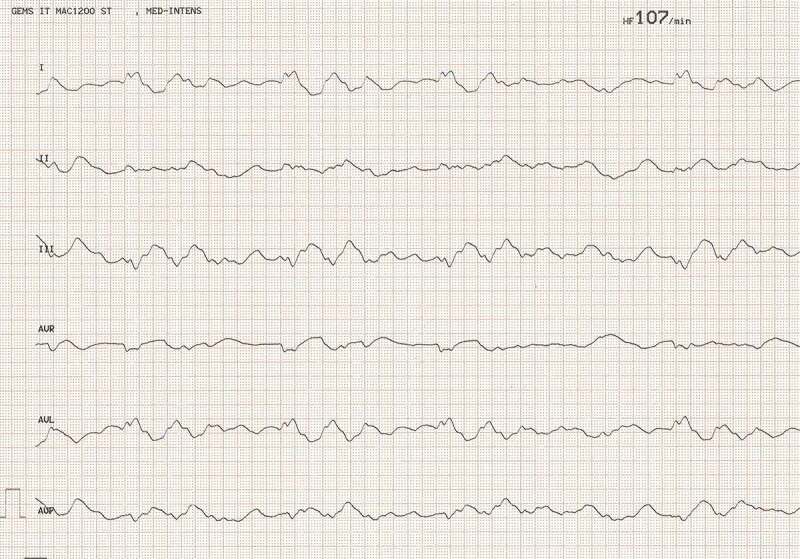
Electrocardiogram after yew poisoning showing an irregular arrhythmia at a rate of 107/min with bizarre QRS prolongation.

**Figure 2.**
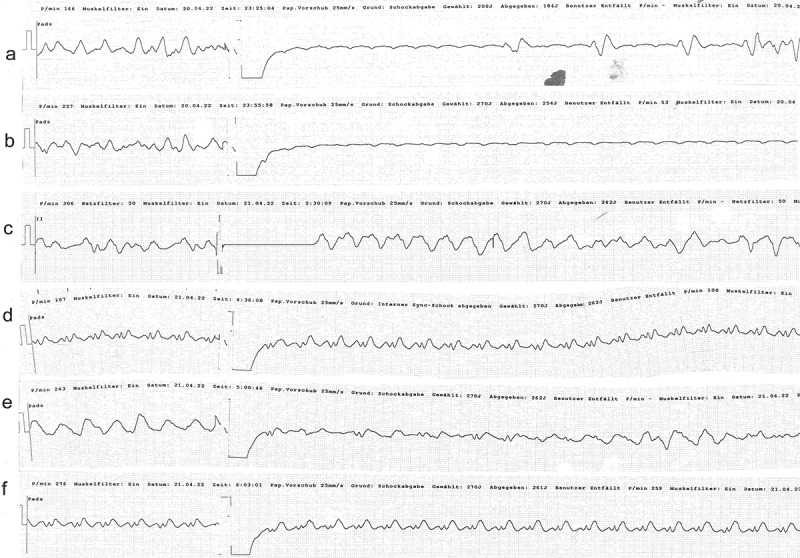
Ineffective defibrillation after ingestion of *Taxus baccata* (a) Irregular wide complex tachycardia terminating to arterial flutter without ventricular response. Seconds later, broad ventricular complexes appear that quickly degenerate into the irregular wide complex tachycardia. (b) Irregular wide complex tachycardia terminating to arterial flutter without ventricular response. (c-f) Ineffective defibrillation of wide complex tachycardia.

**Figure 3.**
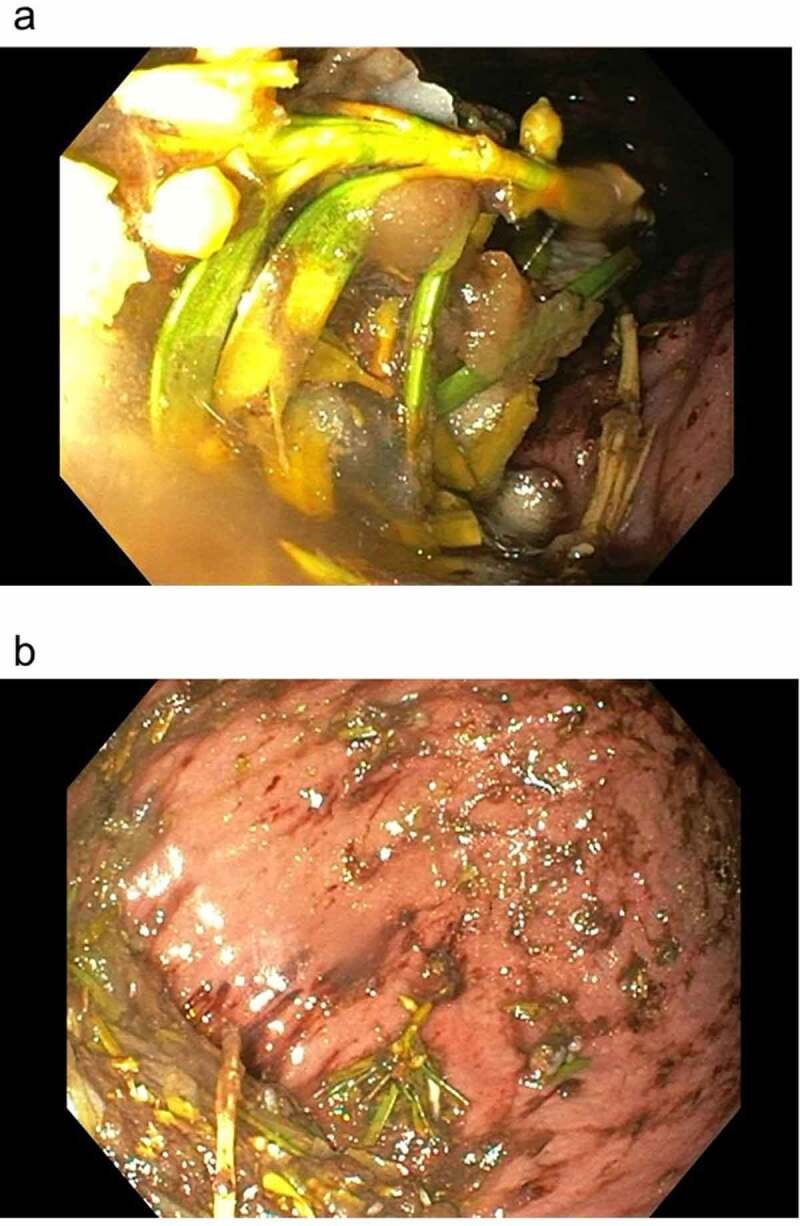
Gastroscopy showing copious foliage of *Taxus baccata.*

**Figure 4.**
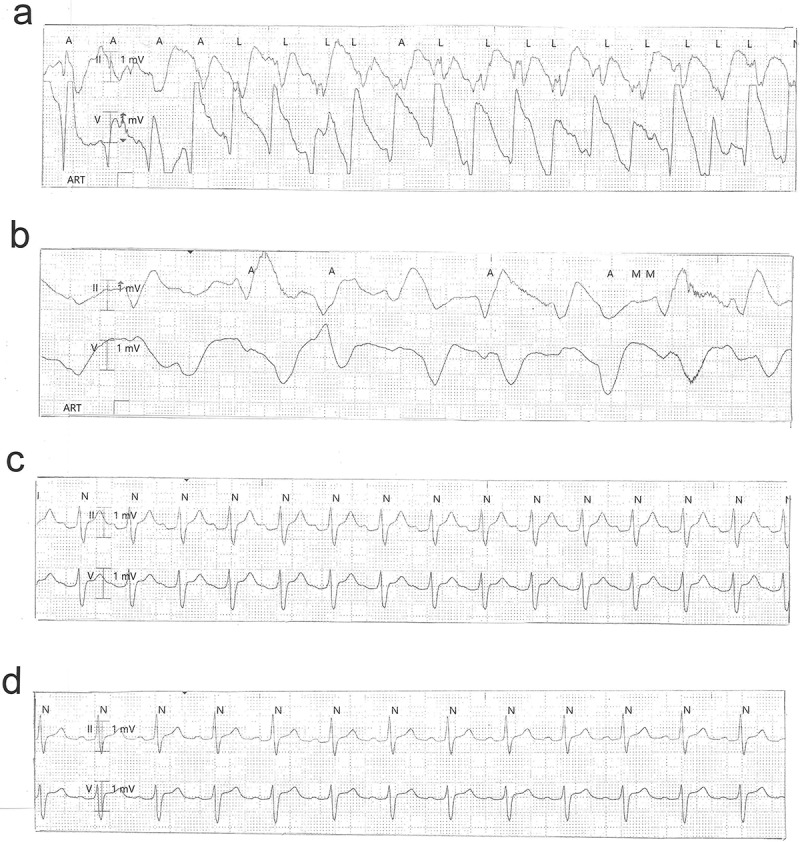
Sequential electrocardiograms 2 ½ h (a) 3 h (b), 18 h (c), and 22 h (d) after ingestion of *Taxus baccata.*

The 39-year old patient presented to our emergency department two hours after having deliberately ingested approximately 30 g of *Taxus baccata* leaves and branches. He had collapsed in his residential home for psychiatric patients, clarified himself that he had ingested yew in a suicidal attempt, and was brought to our hospital by emergency medical services. His past medical history included schizophrenia and chronic hepatitis C. His premedication consisted of zolpidem, sertraline, olanzapine, clozapine, trazodone, oxazepam, and paliperidon. On arrival in the emergency department, he was somnolent (Glasgow coma scale 8), had a heart rate of 117/min, an oxygen saturation of 95%, and a body temperature of 36.9°C. A faint peripheral pulse was palpable but no blood pressure readings could be obtained. He was immediately transferred to ICU. Five minutes after arrival on the ICU, we started with cardiopulmonary resuscitation because of pulseless electric activity. During cardiopulmonary resuscitation, we administered adrenaline (4 mg), esketamine (250 mg), midazolam (5 mg), rocuronium (100 mg), magnesium (4.8 mmol), sodium bicarbonate 8.4% (100 ml), balanced crystalloid fluid (1500 ml), and noradrenaline (0.70 µg/kg/min). The initial electrocardiogram showed an irregular arrhythmia at a rate of 107/min with bizarre (non-typical bundle branch block) QRS prolongation >600 ms followed by irregular tachy- as well as bradyarrhythmias (Figure 1). Emergency echocardiography during cardiopulmonary resuscitation showed no pericardial effusion, and no relevant cardiac contractions. Defibrillation was ineffective (Figure 2). As there is no validated antidote for yew intoxication, we immediately performed extracorporeal life support via VA-ECMO, which was implanted during ongoing resuscitation with an automated chest compression system (LUCAS-2, Jolife AB, Lund Sweden). We used a 23 Fr drainage cannula in the left femoral vein and a 19 Fr return cannula in the right femoral artery with an antegrade perfusion catheter. Cardiopulmonary resuscitation with chest compressions was done for 39 minutes. In this period, we managed to perform tracheal intubation, central venous access, arterial line, emergency echocardiography, (ineffective) defibrillation, and implantation of VA-ECMO as a rescue therapy. Lactic acid levels rose to a maximum of 4.4 mmol/L 35 min after arrival on ICU and rapidly turned normal with start of VA-ECMO. It was not necessary to introduce a left ventricular assist device. The initial laboratory analysis showed a high white blood cell count of 25 G/l, a normal C-reactive protein of 0.8 mg/dl (normal range <5 mg/dl), and slightly elevated aspartate and alanine aminotransferase levels of 145 and 87 U/l, respectively (normal range < 50 U/l). We next conducted a gastroscopy, which revealed plenty of ingested yew leaves and branches (Figure 3). Gastric juice was removed together with the copious foliage (using endoscopic baskets), and activated charcoal was given via a nasogastric tube to reduce further resorption of toxins. Cardiac electrophysiology slowly recovered within the next hours, as shown in the sequential electrocardiograms (Figure 4). The initial pH of 7.372 decreased to a nadir of 7.276 after 10 hours and rapidly recovered to normal ranges thereafter. High-sensitivity cardiac troponin T levels reached a maximum of 161 pg/ml on day 2 (normal range <14 pg/ml), and N-terminal pro-B-type natriuretic peptide (NT-proBNP) was slightly elevated to 134 pg/ml (normal range <100 pg/ml). Echocardiography on day 2 showed a left ventricular ejection fraction of 40%, no regional hypokinesis, and no pericardial effusion. Therefore, VA-ECMO support was stopped after 39 hours, and vascular surgeons removed cannulas. We liberated the patient from mechanical ventilation after seven days. He had no neurological deficit, and perfectly recalled what had happened. Neuronal specific enolase was within reference range, indicating that no relevant cerebral ischemia was present throughout treatment. On day 7, echocardiography showed full cardiac recovery with normal diameters of the cardiac chambers, normal left ventricular ejection fraction (60%), and normal right ventricular function (tricuspid annular plane systolic excursion 25 mm). The patient was discharged from ICU to psychiatry after 12 days and was dismissed from hospital after 23 days.

## Discussion

Cardiotoxicity of the European yew derives from a mixture of the major alkaloids taxine A and B, which block cardiac voltage-gated sodium and calcium currents [2]. Taxines are contained in most parts of the *Taxus baccata* including the bark, leaves, and the hard central seed, whereas the surrounding fleshy red pulp, the aril, does not contain any toxins. The lethal oral dose of yew leaves was estimated to be in the range of 0.6 to 1.3 g yew leaves/kg body weight [2]. Clinical manifestations of yew intoxication often begin with abdominal discomfort, nausea, dizziness, and vomiting starting within a few hours after exposure [2]. The major clinical hazard of taxine A and B is that they can induce cardiac arrhythmias ranging from atrioventricular conduction blocks to refractory ventricular fibrillation [3–10]. The clinical severity depends on quantity, type as well as route of exposure. Taxine A and B are volatile and difficult to detect. Therefore, paclitaxel can be used as a specific biomarker for yew poisoning in primary high performance liquid chromatography with mass spectrometry (HPLC-MS) screening, when patient history and gastroscopy are inconclusive [11].

A literature search on PubMed using the mesh-terms ECMO AND (taxine OR yew OR taxus) revealed a handful of other case reports published involving patients which were resuscitated using VA-ECMO after intentional ingestion of yew leaves and branches leading to cardiogenic shock [3–10]. Additional therapeutic approaches included albumin dialysis, digoxin immune Fab, and lipid-emulsion therapy [8,10,12,13]. However, these approaches are not feasible in patients undergoing cardiopulmonary resuscitation.

Thus, the present report strengthens the notion that prompt VA-ECMO can provide a bridging to full recovery for patients with yew poisoning and cardiogenic shock [3–10], and emphasizes the role of intoxications as reversible causes of cardiac arrest. The impressive ineffectiveness of defibrillation and/or overdrive pacing against taxine-derived arrhythmias is in line with prior publications [4,14,15]. The outcome of patients with acute cardiogenic shock due to yew poisoning treated with VA-ECMO appears to be excellent and the present case adds to the body of existing literature encouraging the early use of VA-ECMO in these cases. Prompt primary toxin-elimination using endoscopic tools and activated charcoal reduce toxin resorption and thus may shorten the necessary time of extracorporeal cardiopulmonary support in critically ill patients.

## Data Availability

The data that support the findings of this study are available on request from the corresponding author, [PE], upon reasonable request. The data are not publicly available due to privacy restrictions.
